# Vitamin D3 Exerts Beneficial Effects on C2C12 Myotubes through Activation of the Vitamin D Receptor (VDR)/Sirtuins (SIRT)1/3 Axis

**DOI:** 10.3390/nu15224714

**Published:** 2023-11-07

**Authors:** Nurul Fatihah Talib, Zunshu Zhu, Kyoung-Soo Kim

**Affiliations:** 1 Department of Biomedical Science, Graduate School, Kyung Hee University, Seoul 02447, Republic of Korea; nfatihaht96@gmail.com (N.F.T.); zzyiwnl06@naver.com (Z.Z.); 2 Department of Clinical Pharmacology and Therapeutics, Kyung Hee University School of Medicine, Seoul 02447, Republic of Korea; 3 East-West Bone & Joint Disease Research Institute, Kyung Hee University Hospital at Gangdong, Seoul 05278, Republic of Korea

**Keywords:** vitamin D, vitamin D receptor (VDR), sirtuins, sarcopenia, muscle atrophy, mitochondrial biogenesis, oxidative phosphorylation (OXPHOS)

## Abstract

The onset of sarcopenia is associated with a decline in vitamin D receptor (VDR) expression, wherein reduced VDR levels contribute to muscle atrophy, while heightened expression promotes muscle hypertrophy. Like VDR, the age-related decline in protein deacetylase sirtuin (SIRT) expression is linked to the development of sarcopenia and age-related muscle dysfunction. This study aimed to investigate whether the VDR agonist 1,25-dihydroxyvitamin D3 (1,25VD3) exerts beneficial effects on muscles through interactions with sirtuins and, if so, the underlying molecular mechanisms. Treatment of 1,25VD3 in differentiating C2C12 myotubes substantially elevated VDR, SIRT1, and SIRT3 expression, enhancing their differentiation. Furthermore, 1,25VD3 significantly enhanced the expression of key myogenic markers, including myosin heavy chain (MyHC) proteins, MyoD, and MyoG, and increased the phosphorylation of AMPK and AKT. Conversely, VDR knockdown resulted in myotube atrophy and reduced SIRT1 and SIRT3 levels. In a muscle-wasting model triggered by IFN-γ/TNF-α in C2C12 myotubes, diminished VDR, SIRT1, and SIRT3 levels led to skeletal muscle atrophy and apoptosis. 1,25VD3 downregulated the increased expression of muscle atrophy-associated proteins, including FoxO3a, MAFbx, and MuRF1 in an IFN-γ/TNF-α induced atrophy model. Importantly, IFN-γ/TNF-α significantly reduced the mtDNA copy number in the C2C12 myotube, whereas the presence of 1,25VD3 effectively prevented this decrease. These results support that 1,25VD3 could serve as a potential preventive or therapeutic agent against age-related muscle atrophy by enhancing the VDR/SIRT1/SIRT3 axis.

## 1. Introduction

Sarcopenia is an age-related condition characterized by decreased skeletal muscle mass and strength [1]. It is a prevalent condition affecting approximately 11–50% of individuals over 80 years of age [2]. As skeletal muscle accounts for a notable fraction (50–75%) of total body proteins, muscle atrophy attributed to sarcopenia poses significant health risks, including frailty, increased risk of falls and accidents, diabetes, and cardiovascular diseases [3]. Unfortunately, there are currently no specific safe pharmacological treatments available for sarcopenia, and while nutritional interventions and exercise are the only recommended safe approaches, the extent of their effectiveness is an area of ongoing research and discussion [4].

Recent studies have highlighted the potential role of vitamin D receptor (VDR) in sarcopenia and muscle health. VDR protein expression is lower in the forearm muscles of sarcopenic patients than in non-sarcopenic patients [5]. The induction of VDR overexpression in the muscle of the rat tibialis results in enhanced muscle protein synthesis (MPS) and increased anabolic signaling, ultimately resulting in hypertrophy [6]. In contrast, reduced VDR expression induces skeletal muscle atrophy [7]. The interaction between the VDR and muscle health is intricately linked to the essential role of vitamin D.

Vitamin D is a fat-soluble vitamin acquired mainly through UVB exposure (90%) and dietary intake (10%). Upon conversion into its active form, 1,25-dihydroxyvitamin D3 (1,25VD3), in the kidney, it acts as a potent agonist of VDR in skeletal muscle, triggering diverse physiological processes [8,9]. Serum vitamin D levels typically decrease with age for several reasons. Older people stay indoors and have less sun exposure to their skin. As the skin synthesis activity of vitamin D production decreases, and as the kidneys age, the conversion of vitamin D to its active form, 1,25VD3, also decreases [10,11]. Vitamin D deficiency ultimately contributes to decreased VDR stimulation and decreases VDR expression with age [10,11]. Older adults with vitamin D deficiency show decreased muscle mass, strength, and performance, which increase the risk of falls. Vitamin D supplementation has been shown to reduce the risk of falls, improve muscle strength and function, and has been shown to increase skeletal muscle fiber size by 10% in older women [12,13]. These findings suggest a positive relationship between vitamin D/VDR and muscle mass and function [14,15].

Sirtuins are a family of protein deacetylases that depend on nicotinamide adenine dinucleotide (NAD+) cofactors. Sirtuins govern various cellular processes, including aging, metabolism, stress responses, and inflammation, and are considered anti-aging interventions [16]. The roles of SIRT1 and SIRT3 in maintaining muscle function and preventing aging have been extensively studied. SIRT1 and SIRT3 specifically regulate mitochondrial activity and biogenesis, oxidative stress, and muscle fiber type switching in muscle tissue [17,18]. Similar to VDR, the expression of SIRT1 and SIRT3 declined with age [19,20]. The age-related decrease in SIRT1 and SIRT3 levels is linked to the development of sarcopenia and age-related muscle dysfunction [21,22].

Exercise has been shown to increase the expression of SIRT1 and SIRT3, consequently improving muscle function. Rat studies have revealed that acute resistance exercise can also elevate VDR expression in skeletal muscle, contributing to muscle hypertrophy [17,23]. An increase in VDR, SIRT1, and SIRT3 expression in response to exercise suggests a potential relationship between these proteins. In addition, several studies have shown that vitamin D treatment increases SIRT1 and SIRT3 expression [24,25]. Consequently, 1,25VD3 has emerged as a potential therapeutic target for sarcopenia, either as an alternative or in combination with exercise interventions [26]. However, further studies are required to understand the mechanisms underlying the beneficial effects of 1,25VD3 supplementation on muscle health.

In this study, we investigated the mechanism underlying the interaction between the 1,25VD3/VDR signaling systems and SIRT1 and SIRT3. We observed that VDR, SIRT1, and SIRT3 form an interdependent relationship, which we refer to as the VDR/SIRT1/SIRT3 axis. Downregulation of VDR leads to decreased signaling in the VDR/SIRT1/SIRT3 axis, resulting in muscle cell atrophy and apoptosis. In addition, 1,25VD3 treatment reversed these detrimental effects such as muscle atrophy and apoptosis induced by Interferon-γ (IFN-γ)/Tumor necrosis factor-α (TNF-α) through this axis. These findings suggest that 1,25VD3 has the potential to be developed as a therapeutic agent against sarcopenia by promoting muscle cell development, maintenance, and protection against atrophy and apoptosis through the activation of VDR, SIRT1, and SIRT3.

## 2. Materials and Methods

### 2.1. Cell Culture

The mouse muscle cell line C2C12 was obtained from ATCC (Manassas, VA, USA) and cultured in a humidified incubator at 37 °C and 5% CO_2_ in a growth medium containing Dulbecco’s modified Eagle’s medium (DMEM) with 4500 mg/L glucose (Gibco, Carlsbad, CA, USA), 10% fetal bovine serum (Gibco), and 1% penicillin-streptomycin (Gibco Life Technologies, Waltham, MA, USA). To induce myogenic differentiation, C2C12 cells were cultured in a 60 mm dish to 90% confluence before switching from the growth medium to the low-serum differentiation medium containing DMEM supplemented with 2% horse serum (Gibco Life Technologies, Waltham, MA, USA) and 1% penicillin-streptomycin The differentiation medium was refreshed every 48 h. After four days of differentiation, C2C12 myotubes were exposed to treatments with varying concentrations of 1,25-dihydroxyvitamin D3 (BML-DM200; Enzo Life Sciences, New York, NY, USA), either individually or in conjunction with Recombinant Mouse TNF-α (aa80-235; R&D Systems, Minneapolis, MN, USA) reconstituted in PBS and Recombinant IFN-γ (R&D Systems, Minneapolis, MN, USA) reconstituted in PBS for 24 h. Subsequently, the diameter of the C2C12 myotubes was measured. Following this, the myotubes underwent either protein or mRNA analysis or mitochondrial DNA copy number analysis.

### 2.2. DsiRNA Transfection

DsiRNA oligonucleotides against VDR and the negative control were purchased from IDT Technology (Integrated DNA Technologies, Iowa City, IA, USA). C2C12 Myotubes that had undergone differentiation for three days were transiently transfected with DsiRNA oligonucleotides for 48 h using Mirus Transit TKO transfection reagents (Mirus Bio LLC, Madison, WI, USA). Subsequently, on the fifth day of differentiation, C2C12 cells were washed twice with cold PBS and then utilized for protein or mRNA analysis. The DsiRNA duplex sequences used to knockdown VDR expression were:

5′-GGAUAUCAGCAUAAACCUAUGUCTG-3′ (forward);
3′-GACCUAUAGUCGUAUUGGAUACAGAC-5′ (reverse).

### 2.3. Quantitative Real-Time PCR Analysis

Total RNA was isolated using the FavorPrep™ Tri-RNA Reagent (Favorgen Biotech Corp, Michigan, MI, USA) according to the manufacturer’s guidelines. cDNA was reverse transcribed from 1 μg of total RNA using the RevertAid First Strand cDNA Synthesis Kit (Thermo Scientific, Waltham, MA, USA). To determine gene expression levels, quantitative real-time PCR was performed using a 7500 Real-Time PCR System (Applied Biosystems, Carlsbad, CA, USA) with ExcelTaq™ 2× Fast Q-PCR Master Mix (SYBR, ROX; Smobio, CA, USA), forward and reverse primers of the cDNA template, and water to a final volume of 20 μL. A list of primers used is provided in Table S1. Relative mRNA expression levels were calculated from the threshold cycle value of each PCR product and normalized to that of β-actin using the comparative threshold cycle method.

### 2.4. Western Blot Analysis

Myotubes were collected and processed for protein analysis using RIPA lysis and extraction buffer (Thermo Scientific, Waltham, MA, USA). Briefly, samples were washed twice with ice-cold PBS, scraped, and then pelleted by centrifugation at 14,000× *g* for 20 min at 4 °C. Protein quantification was performed using a Pierce BCA Protein Assay Kit (Thermo Scientific, Waltham, MA, USA). Proteins were resolved using sodium dodecyl sulfate-polyacrylamide gel electrophoresis and electro-transferred onto nitrocellulose membranes. Membranes were then blocked with 5% (*w*/*v*) skim milk, incubated in primary antibodies overnight, washed with TBS-T, and then incubated in secondary antibodies for 1 h. Protein expression was detected using ECL reagents (DoGenBio, Seoul, Republic of Korea), and band intensities were quantified and normalized to α-Tubulin using ImageJ software 1.50i (NIH, Bethesda, MD, USA). The antibodies used in this study are listed in Table S2. Data are expressed as fold-change relative to the control.

### 2.5. Cell Viability Assays

Cell viability assays were performed using the methyl thiazole tetrazolium (MTT) assay in a 12-well plate. After four days of differentiation, C2C12 myotubes were treated for 24 h with various concentrations of the test compounds: 1,25-dihydroxyvitamin D3 diluted in DMSO; Recombinant Mouse TNF-α reconstituted in PBS, Recombinant IFN-γ reconstituted in PBS. Afterward, 300 μL of MTT solution (5 mg/mL) was added to each well in the growth medium for 3 h at 37 °C in the dark. The MTT solution was removed, and the formazan crystals were dissolved in 1 mL of DMSO/well for 20 min. The optical density of solubilized formazan in each well was quantified spectrophotometrically at 540 nm using a BioTek Synergy HTX Multimode Reader (Agilent Technologies, St. Clara, CA, USA).

### 2.6. Myotube Diameter Measurements

Images of myotubes on the fifth day of differentiation were captured at 100× magnification to determine myotube diameter. Five distinct images were captured from each sample and ten myotubes were randomly selected from each image, and the diameter of each myotube was measured using ImageJ software. The measures were taken on coded photographs in a “blinded” method, with the investigator unaware of the group from which the cultures originated.

### 2.7. Genomic DNA Isolation and Mitochondrial DNA Copy Number Analysis

C2C12 myotubes were trypsinized, centrifuged, and resuspended in 200 µL of 1× PBS. Genomic DNA was extracted and purified using a PureLink Genomic DNA Mini Kit (K1820-00; Invitrogen, Carlsbad, CA, USA) according to the manufacturer’s guidelines. The relative mitochondrial DNA copy number was determined by the ratio of the Ct value of the mitochondrial Cox1 (MTCO1) gene to that of the nuclear β-actin gene, calculated using the following formula: Amplification efficiency^(Ct reference – Ct target)^. Relative gene expression was analyzed using the 2^−ΔΔCT^ method by normalizing to β-actin gene levels.

### 2.8. Statistical Analysis

Data analysis was performed using GraphPad Prism, version 8.0. Experimental results are presented as the mean ± SEM. Statistical significance was determined using the Mann–Whitney U test between the means of two groups or one-way ANOVA for comparison among multiple groups, followed by Tukey’s post hoc test.

## 3. Results

### 3.1. 1,25VD3 Induces C2C12 Myotube Hypertrophy and Increases C2C12 Myogenic Differentiation via Upregulation of VDR, SIRT1 and SIRT3 Expression

**Figure 1:**
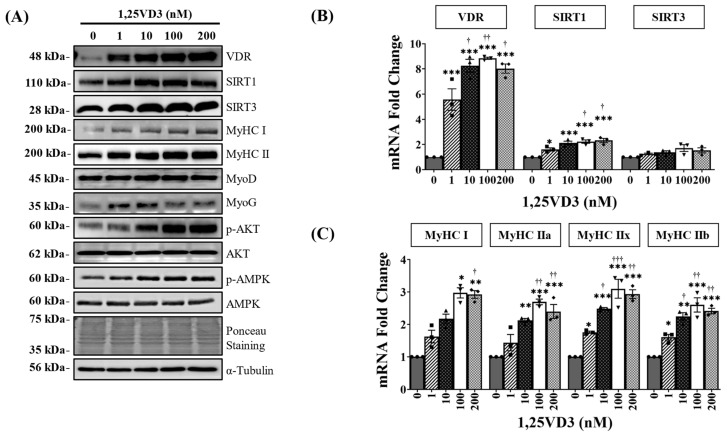
Effects of 1,25VD3 on VDR, SIRT1, SIRT3 expression, myogenic differentiation, and AMPK/AKT activation. C2C12 myotubes were differentiated for four days and treated with various concentrations of 1,25VD3 (0 nM, 1 nM, 10 nM, 100 nM, 200 nM) for 24 h. (**A**) Representative Western blotting images of VDR, SIRT1, SIRT3, MyHC proteins (MyHC I and II), myogenic markers (MyoD and MyoG), p-AMPK, AMPK, p-AKT, and AKT. (**B**) qPCR analysis of the VDR, SIRT1, and SIRT3 mRNA levels. (**C**) qPCR analysis of MyHC I, IIa, IIx and IIb mRNA levels. The symbols in (**B**,**C**) serve as points to represent values. Different shapes are used to visually distinguish the values for each group. The data are representative of three independent experiments and are presented as the mean ± SEM. One-way ANOVA was used for statistical analysis between the negative control and 1,25VD3-treated groups. Statistical significance is represented as * *p* < 0.05, ** *p* < 0.01, and *** *p* < 0.001 when compared with 0 nM (control group), and † *p* < 0.05, †† *p* < 0.01, and ††† *p* < 0.001 when compared to 1 nM group. See Supplementary Figure S1 for myotube diameter measurements and Western blot quantifications.

To investigate the hypertrophic effects of 1,25VD3 on normal muscle cells, C2C12 myoblasts were differentiated for four days and then treated with varying concentrations of 1,25VD3 for a duration of 24 h. The results revealed a significant increase in the protein and mRNA expression of VDR, SIRT1, and SIRT3 following 1,25VD3 treatment (Figure 1A,B and Figure S1b). Further investigation using Western blotting and RT-PCR confirmed that 1,25VD3 increased the levels of Myosin heavy chain (MyHC) proteins (MyHC I and II) and mRNA (MyHC I, IIa, IIx, and IIb), indicating its hypertrophic effect (Figure 1A,C and Figure S1c). The induction of MyoD and MyoG protein expression further supported the positive role of 1,25VD3 in promoting myogenic differentiation (Figure 1A and Figure S1d). Moreover, 1,25VD3 also stimulates the activation of AMP-activated protein kinase (AMPK) and AKT (protein kinase B) (Figure 1A and Figure S1e). These findings suggest that the 1,25VD3-induced increase in VDR, SIRT1, and SIRT3 levels may promote muscle cell development and growth in C2C12 myotubes as evidenced by the increase in myotube diameters (Figure S1a). These effects are likely mediated by the activation of downstream targets such as AMPK and AKT.

### 3.2. Transient VDR Knockdown Downregulates SIRT1 and SIRT3 Expression and Induces Myotube Atrophy

**Figure 2:**
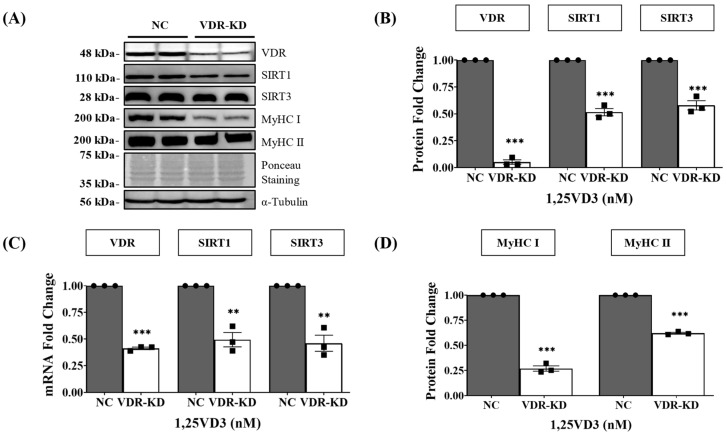
Effects of transient VDR knockdown in C2C12 myotubes on VDR, SIRT1, SIRT3, and MyHC expression. C2C12 myotubes were differentiated by serum depletion and transfected with VDR DsiRNA on the third day of differentiation for 48 h. (**A**) Representative Western blotting images of VDR, SIRT1, SIRT3, and MyHC (MyHC I and II). (**B**) Western blot quantification of VDR, SIRT1, and SIRT3 expression. (**C**) qPCR analysis of the VDR, SIRT1, and SIRT3 mRNA levels. (**D**) Western blot quantification of MyHC I and MyHC II expressions. The symbols in (**B**–**D**) serve as points to represent values. Different shapes are used to visually distinguish the values for each group. The data are representative of three independent experiments Mann–Whitney U test was used for statistical analysis between the negative control (NC) and VDR-KD (VDR knockdown) groups. Statistical significance is represented by ** *p* < 0.01, and *** *p* < 0.001.

We further investigated whether VDR expression is closely linked to SIRTs expression and myotube development in C2C12 myotubes. Transfection with VDR DsiRNA resulted in a ~95% decrease in VDR protein levels (*p* < 0.001) in VDR-knockdown (KD) myotubes. SIRT1 protein expression was reduced by 48% (*p* < 0.001) in these myotubes, whereas SIRT3 protein expression was decreased by 42% (*p* < 0.001) (Figure 2A,B). These data were further supported by qPCR analysis (Figure 2C). Downregulation of VDR in VDR-KD myotubes resulted in a significant reduction in SIRT1 and SIRT3 expression, which led to a decrease in MyHC protein (MyHC I and II) expression, supporting the notion that downregulation of VDR, SIRT1, and SIRT3 induces myotube atrophy (Figure 2A,D). Downregulation of SIRT1 and SIRT3 expression and the concomitant reduction in MyHC expression in VDR-KD myotubes indicate the function of the VDR/SIRT1/SIRT3 axis in myogenesis.

### 3.3. IFN-γ/TNF-α Treatment Induces Atrophy and Apoptosis in C2C12 Myotubes via Downregulation of VDR/SIRT1/SIRT3 Axis

**Figure 3:**
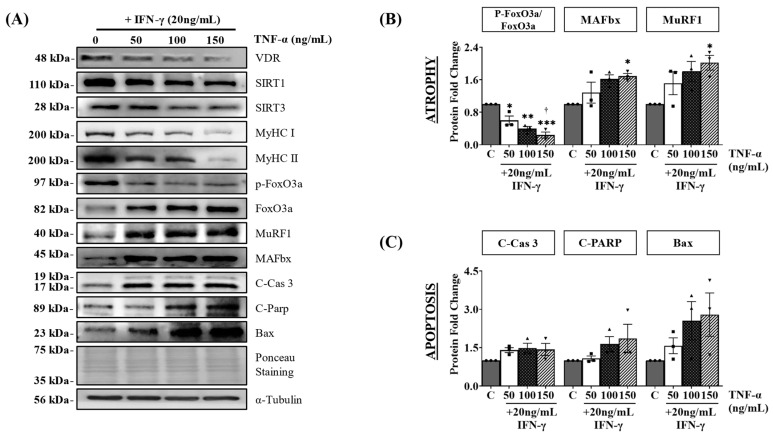
Effects of IFN-γ/TNF-α-induced VDR, SIRT1, and SIRT3 downregulation on myotube atrophy and apoptosis. After four days of differentiation, C2C12 myotubes were exposed to various concentrations of IFN-γ/TNF-α, including control (PBS) and 20 ng/mL IFN-γ combined with 50, 100, or 150 ng/mL TNF-α for 24 h. (**A**) Representative Western blot images of VDR, SIRT1, SIRT3, MyHC (MyHC I and II), atrophy markers (FoxO3a, MuRF1, and MAFbx), and apoptosis markers (cleaved caspase-3, cleaved PARP, and Bax). (**B**) Quantification of atrophy markers (pFoxO3a/FoxO3a, MAFbx, and MuRF1). (**C**) Quantification of apoptosis-related proteins (cleaved caspase-3, cleaved PARP, and Bax). The expression of phosphorylated FoxO3a (p-FoxO3a) was normalized to that of total FoxO3a, whereas the expression of other proteins was normalized to that of α-tubulin. The symbols in (**B**,**C**) serve as points to represent values. Different shapes are used to visually distinguish the values for each group. The data are representative of three independent experiments. One-way ANOVA was used for statistical analysis between the negative control and IFN-γ/TNF-α treated groups. Statistical significance is represented as * *p* < 0.05, ** *p* < 0.01, and *** *p* < 0.001 when compared to the control group, and † *p* < 0.05 when compared to the 20 ng/mL IFN-γ + 50 ng/mL TNF-α group. Refer to Supplementary Figure S2 for Western blot quantification of VDR, SIRT1, SIRT3, MyHC I, and MyHC II.

Co-treatment of C2C12 myotubes with IFN-γ and TNF-α is a typical in vitro atrophy model used to study sarcopenia, which is associated with chronic muscular inflammation [27]. In the present study, we used this model to examine the association between the VDR/SIRT1/SIRT3 axis and muscle health. C2C12 myotubes were differentiated for four days and treated for 24 h with various doses of TNF-α (0, 50, 100, or 150 ng/mL) in combination with 20 ng/mL IFN-γ. Protein expression analysis revealed a significant decrease in VDR protein expression in myotubes treated with IFN-γ/TNF-α, accompanied by a reduction in SIRT1 and SIRT3 protein expression (Figure 3A and Figure S2a). Moreover, IFN-γ/TNF-α co-treatment led to a substantial decrease in MyHC (I and II) protein expression, indicating impaired myotube formation and increased atrophy (Figure 3A and Figure S2b). Additionally, our findings demonstrated the upregulation of muscle atrophy-associated proteins including FoxO3a, MAFbx, and MuRF1. Notably, a decrease in FoxO3a phosphorylation was observed, suggesting enhanced nuclear translocation and increased FoxO3 transcriptional activity (Figure 3A,B). Furthermore, increased levels of apoptosis-related proteins (cleaved caspase-3, cleaved PARP, and Bax) indicated the induction of apoptosis in C2C12 myotubes following IFN-γ/TNF-α co-treatment (Figure 3C).

### 3.4. 1,25VD3 Ameliorates Myotube Atrophy and Apoptosis Induced by IFN-γ/TNF-α Co-Treatment

**Figure 4:**
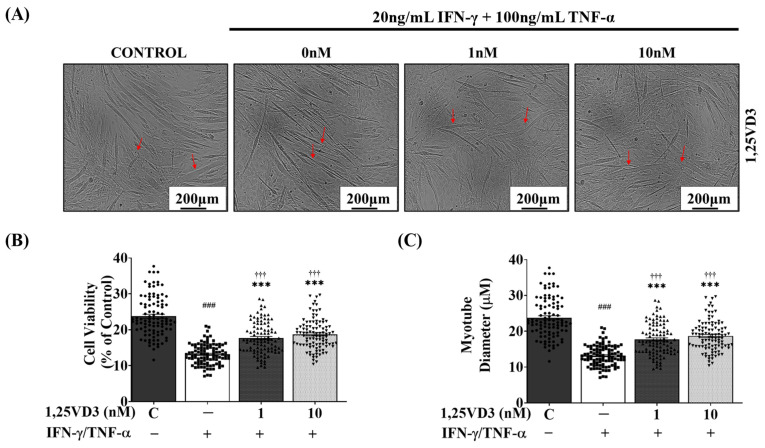
Effects of 1,25VD3 treatment on myotube diameter and cell viability in IFN-γ/TNF-α-treated C2C12 myotubes. Differentiated C2C12 myotubes were treated with PBS, 20 ng/mL IFN-γ + 100 ng/mL TNF-α, or 20 ng/mL IFN-γ + 100 ng/mL TNF-α in combination with 1 nM or 10 nM 1,25VD3 for 24 h. (**A**) Representative phase-contrast images (100×) of C2C12 myotubes after 24 h of treatment with IFN-γ/TNF-α with or without 1,25VD3 (scale bar = 200 μm). Red arrows show the changes in the myotube diameter between the groups. (**B**) Measurement of the myotube diameter. (**C**) Cell viability assay (MTT). The data are representative of three independent experiments. The symbols in (**B**,**C**) serve as points to represent values. Different shapes are used to visually distinguish the values for each group. One-way ANOVA was used for statistical analysis between the negative control and 1,25VD3-treated groups. Statistical significance is represented as *** *p* < 0.001 when compared to the control group, ††† *p* < 0.001 when compared to the IFN-γ/TNF-α group, and ### *p* < 0.001 when compared to all other groups.

To investigate whether 1,25VD3 exerted protective effects against atrophy and apoptosis in IFN-γ/TNF-α-treated myotubes, differentiated C2C12 myotubes were either exposed to IFN-γ/TNF-αalone (20 ng/mL + 100 ng/mL) or co-treated with IFN-γ/TNF-α along 1 nM or 10 nM 1,25VD3 for 24 h. 1,25VD3 treatment significantly mitigated the reduction in myotube diameter induced by IFN-γ/TNF-α (Figure 4A,B). Additionally, 1,25VD3 treatment significantly inhibited IFN-γ/TNF-α-induced apoptosis, as indicated by an increase in cell viability (Figure 4C). These results highlight the potential of 1,25VD3 to protect against atrophy and apoptosis in myotubes exposed to IFN-γ/TNF-α.

### 3.5. 1,25VD3 Protects against IFN-γ/TNF-α-Induced Muscle Cell Apoptosis and Atrophy by Upregulating the VDR/SIRT1/SIRT3 Axis and Its Downstream Targets

**Figure 5:**
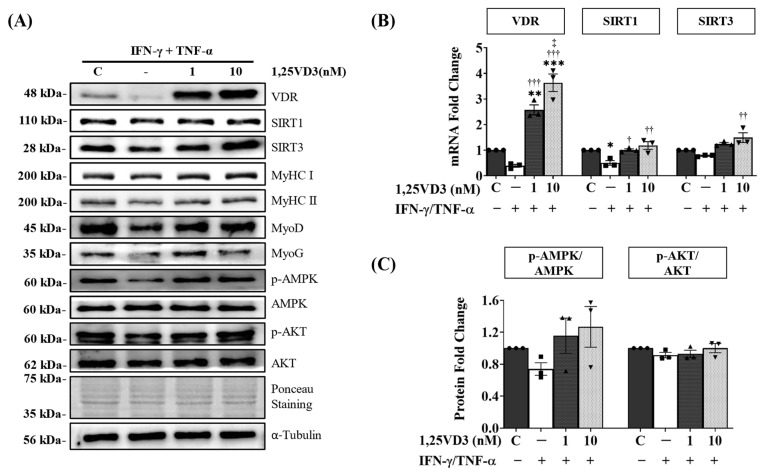
Effects of 1,25VD3 on the rescue of C2C12 myotubes from apoptosis and atrophy by modulation of the downstream targets of VDR, SIRT1, and SIRT3. (**A**) Representative Western blotting images of VDR, SIRT1, SIRT3, MyHC (MyHC I and II), MyoD, MyoG, p-AMPK, AMPK, p-AKT, and AKT expression levels. (**B**) qPCR analysis of VDR, SIRT1, and SIRT3 mRNA levels; (**C**) Western blot analysis of p-AMPK/AMPK and p-AKT/AKT. The symbols in (**B**,**C**) serve as points to represent values. Different shapes are used to visually distinguish the values for each group. The data are representative of three independent experiments. One-way ANOVA was used for statistical analysis and statistical significance is represented as * *p* < 0.05, ** *p* < 0.01, and *** *p* < 0.001 when compared to the control group, † *p* < 0.05, †† *p* < 0.01, and ††† *p* < 0.001 when compared to the IFN-γ/TNF-α group, and ‡ *p* < 0.05 when compared to IFN-γ/TNF-α + 1,25VD3 (1nM) group. Refer to Supplementary Figure S3 for Western blot quantification of VDR, SIRT1, SIRT3, MyHC I, MyHC II, MyoD, and MyoG.

To understand the mechanism underlying the protective effects of 1,25VD3, we investigated the impact of 1,25VD3 treatment on the expression of VDR, SIRT1, and SIRT3. Our findings demonstrated that the decreased levels of VDR, SIRT1, and SIRT3 protein and mRNA expression caused by IFN-γ/TNF-α treatment were effectively restored by 1,25VD3 treatment (Figure 5A,B and Figure S3a). Furthermore, treatment with 1,25VD3 significantly enhanced the expression of key myogenic markers, including MyHC (MyHC I and II), MyoD, and MyoG, compared to the group treated with IFN-γ/TNF-α alone (Figure 5A and Figure S3b,c). These results suggest that the upregulation of the VDR/SIRT1/SIRT3 axis by 1,25VD3 promotes myogenic differentiation and protection against atrophy and apoptosis induced by IFN-γ/TNF-α.

Furthermore, we assessed the expression of AMPK and AKT, which are common targets of VDR, SIRT1, and SIRT3. Our results revealed that IFN-γ/TNF-α treatment reduced AMPK and AKT phosphorylation. However, treatment with 1,25VD3 effectively restored the phosphorylation levels of AMPK and AKT, indicating their activation (Figure 5C). These findings suggest that the VDR/SIRT/SIRT3 axis may exert its protective effects on IFN-γ/TNF-α-induced apoptosis and atrophy, at least in part, through the activation of AMPK and AKT signaling pathways.

### 3.6. Upregulation of VDR/SIRT1/SIRT3 Axis by 1,25VD3 Inhibits Atrophy and Apoptosis in C2C12 Myotubes

**Figure 6:**
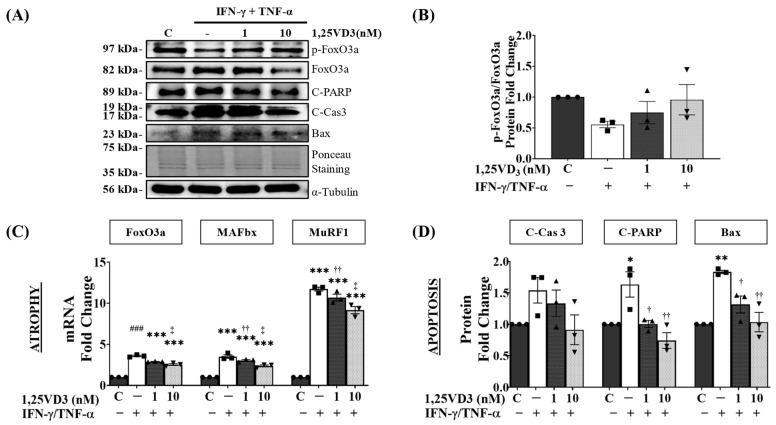
Effects of 1,25VD3 on the rescue of C2C12 myotubes from apoptosis and atrophy. (**A**) Western blot analysis of atrophy markers (pFoxO3a/FoxO3a) and apoptosis markers (cleaved PARP, cleaved caspase 3, and Bax). (**B**) Western blotting quantification of phosphorylated FoxO3a normalized to that of FoxO3a (**C**). qPCR analysis of atrophy-related genes (FoxO3a3a, MuRF1, and MAFbx). (**D**) Quantification of apoptosis-related proteins (cleaved caspase-3, cleaved PARP, and Bax). The symbols in (**B**–**D**) serve as points to represent values. Different shapes are used to visually distinguish the values for each group. The data are representative of three independent experiments. One-way ANOVA was used for statistical analysis. Statistical significance is represented as * *p* < 0.05, ** *p* < 0.01, and *** *p* < 0.001 when compared to the control group, † *p* < 0.05 and †† *p* < 0.01 when compared to the IFN-γ/TNF-α group, ### *p* < 0.001 when compared to all other groups, and ‡ *p* < 0.05 when compared to IFN-γ/TNF-α + 1,25VD3 (1nM) group.

To investigate the signaling pathways involved in the protective effects of 1,25VD3 against the decrease in myotube diameter and cell viability induced by IFN-γ/TNF-α, we examined the expression of key proteins associated with apoptosis and atrophy (Figure 6A). 1,25VD3 administration increased FoxO3a phosphorylation, which was reduced by IFN-γ/TNF-α treatment (Figure 6B). This inhibited FoxO3a translocation into the nucleus and reduced the transcription of FoxO3a and MuRF1/MAFbx pathway-related genes, as evidenced by a decrease in the transcriptional activity of FoxO3a, MuRF1, and MAFbx as observed by real-time PCR (Figure 6C). Additionally, 1,25VD3 + IFN-γ/TNF-α treatment lowered the expression levels of cleaved caspase-3, cleaved PARP, and Bax, which were elevated by IFN/TNF (Figure 6D).

### 3.7. 1,25VD3 Increased Oxidative Phosphorylation Capacity in IFN-γ/TNF-α-Treated Myotubes

**Figure 7:**
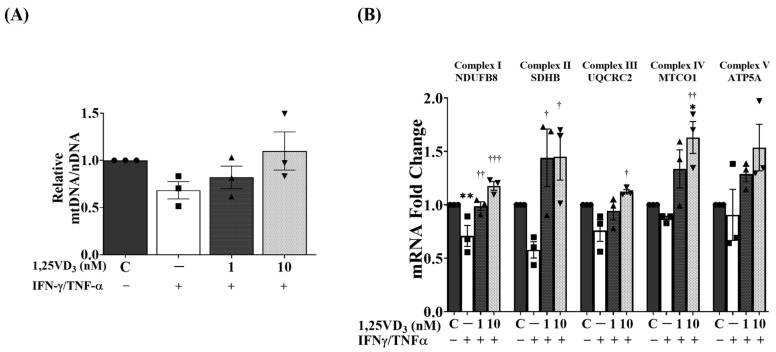
Effects of 1,25VD3 on the augmentation of oxidative phosphorylation capacity in myotubes treated with IFN-γ/TNF-α. (**A**) qPCR analysis of relative mitochondrial DNA normalized to nuclear DNA. (**B**) qPCR analysis of mitochondrial oxidative phosphorylation-related mRNA expression (NDUFB8, SDHB, MTCOI, UQCR2, and ATP5A). The symbols in (**A**,**B**) serve as points to represent values. Different shapes are used to visually distinguish the values for each group. The data are representative of three independent experiments. One-way ANOVA was used for statistical analysis, and statistical significance is represented as * *p* < 0.05 and ** *p* < 0.01 when compared to the control group, and † *p* < 0.05, †† *p* < 0.01, and ††† *p* < 0.001 when compared to the IFN-γ/TNF-α group.

Finally, we assessed the mitochondrial DNA (mtDNA) copy number in the C2C12 myotubes by examining the relative mtDNA-to-nDNA ratio. Our results demonstrated that co-treatment with IFN-γ/TNF-α significantly reduced the mtDNA copy number, whereas the presence of 1,25VD3 effectively prevented this decrease (Figure 7A). Furthermore, we observed that the combined treatment of 1,25VD3 and IFN-γ/TNF-α resulted in an upregulation of mRNA expression for key components of oxidative phosphorylation (OXPHOS) complexes, including NDUFB8, SDHB, MTCO1, UQCR2, and ATP5A (Figure 7B). These findings suggest that 1,25VD3 plays a crucial role in preserving mtDNA copy number and enhancing mitochondrial function in the presence of IFN-γ/TNF-α, thereby potentially mitigating muscle dysfunction.

## 4. Discussion

Numerous studies have explored the potential of exercise or vitamin D supplementation to enhance skeletal muscle health by upregulating VDR, SIRT1, and SIRT3 [6,23,28,29,30]. However, these studies were conducted independently, and none have investigated the combined expression of VDR, SIRT1, and SIRT3 in relation to muscle hypertrophy. Our study investigated the effects of 1,25VD3, an active form of Vitamin D on C2C12 muscle cells and explored the involvement of sirtuins, specifically SIRT1 and SIRT3, in mediating these effects. We found that 1,25VD3 induced hypertrophy in C2C12 myotubes, as shown by the increased expression of MyHC and the myogenic differentiation markers MyoD and MyoG (Figure 1). This suggests that 1,25VD3 promotes muscle development and enhances myogenic differentiation, supporting previous findings regarding the role of 1,25VD3 in increasing VDR expression and skeletal muscle hypertrophy [6,13].

SIRT1 and SIRT3 are NAD+-dependent deacetylases that play crucial roles in gene expression, mitochondrial function, oxidative stress response, and metabolism [18]. Exercise increases SIRT1 and SIRT3 expression, likely by increasing cellular NAD+ levels [17], and also enhances VDR expression [23,31]. Considering the existing data on the positive effects of exercise on skeletal muscle health, particularly in relation to the VDR, SIRT1, and SIRT3, we hypothesized that there may be an interconnected axis involving these factors. In this study, we showed that treatment with 1,25VD3 resulted in a concentration-dependent increase in VDR, SIRT1, and SIRT3 levels (Figure 1). 

Previous studies have demonstrated that VDR and SIRT1 have both direct and indirect connections. Direct interactions occur when the VDR binds to the SIRT1 promoter or to other proteins in the transcription complex. Additionally, VDR and SIRT1 regulate each other via epigenetic modifications [24,30,32,33,34]. Furthermore, 1,25VD3 upregulates VDR and SIRT3 expression in cardiac cells [35]. These findings highlighted the interconnectedness of 1,25VD3/VDR and SIRT1 and SIRT3 in various cellular processes.

The upregulation of VDR, SIRT1, and SIRT3 induced by 1,25VD3 also led to increased phosphorylation of AMPK and AKT (Figure 1). AMPK serves as a crucial regulator of cellular energy metabolism and influences processes, such as glucose absorption, fatty acid oxidation, and mitochondrial biogenesis [36]. AKT plays pivotal roles in protein synthesis, growth, metabolism, and cell survival [37]. The observed increase in AMPK and AKT phosphorylation suggests a regulatory mechanism connecting the VDR, SIRT1, and SIRT3 pathways to AMPK and AKT, underscoring the significance of the VDR/SIRT1/SIRT3 axis in various cellular processes related to skeletal muscle development. However, the significance of AMPK in muscle function is unclear, as several studies have reported contradictory results. Increased AMPK appeared to improve muscle protein production in our in vitro model, most likely due to the involvement of sirtuins. Studies have also shown that when AMPK is activated by exercise, it collaborates with SIRT1 to enhance muscle growth [38]. However, it is worth noting that some studies have suggested that AMPK activation may potentially counteract muscle hypertrophy, highlighting its complex role in regulating muscle mass. During periods of energy deficiency or cellular stress, AMPK is activated to conserve energy, potentially leading to muscle atrophy [39,40]. This underscores the nuanced nature of AMPK’s role of AMPK in muscle physiology, which is influenced by specific contexts, signaling pathways, and metabolic states.

To understand the role of VDR in the VDR/SIRT1/SIRT3 axis in muscle cells, we knocked down VDR expression in C2C12 myotubes. Our findings support an interdependent relationship between VDR, SIRT1, and SIRT3, with VDR likely acting as an upstream regulator that influences the expression and activity of these sirtuins. Muscle atrophy and reduced MyHC expression upon VDR knockdown highlight the importance of VDR in maintaining muscle health (Figure 2). Moreover, VDR deletion has detrimental effects on skeletal muscle cells in vivo leading to muscle atrophy [7,41]. VDR silencing in C2C12 myotubes and G8 murine myoblast cell lines also reduces MyoD and MyoG expression, leading to the inhibition of myogenic differentiation [42]. Further investigation into the expression of SIRT1 and SIRT3 could provide additional evidence regarding their roles in the VDR/SIRT1/SIRT3 axis and their involvement in skeletal muscle myogenesis in vitro.

IFN-γ and TNF-α are pro-inflammatory cytokines that cause muscle atrophy by promoting inflammation, disrupting muscle balance, disrupting myogenesis, and inducing apoptosis [43]. Studies have shown higher levels of IFN-γ and TNF-α in sarcopenic patients compared to non-sarcopenic individuals [44,45]. An in vitro atrophy model using IFN-γ/TNF-α co-treatment in C2C12 myotubes allows the investigation of muscle atrophy mechanisms and the therapeutic effects of 1,25VD3. Our study revealed that IFN-γ/TNF-α co-treatment reduces the expression of VDR, SIRT1, and SIRT3, leading to increased apoptotic cell death and muscle atrophy as observed in a decrease in cell viability and myotube diameter (Supplementary Figure S2). Moreover, it activates FoxO3a transcription factors, resulting in the elevated expression of muscle-specific E3 ubiquitin ligases MAFbx and MuRF1, which contribute to protein degradation [46]. The observed increase in cleaved-caspase 3, cleaved-Parp, and Bax expression further supports the impact of IFN-γ/TNF-α co-treatment on apoptotic cell death (Figure 3). These findings underscore the detrimental impact of IFN-γ/TNF-α co-treatment on muscle health and emphasize the importance of VDR, SIRT1, and SIRT3 in regulating muscle homeostasis and preventing muscle atrophy.

Using this atrophy model, we examined the therapeutic effects of 1,25VD3 in C2C12 myotubes and found that it effectively rescued the myotubes from IFN-γ/TNF-α-induced apoptosis and atrophy, leading to increased cell viability and myotube diameter compared to IFN-γ/TNF-α treatment alone (Figure 4). 1,25VD3 treatment on IFN-γ/TNF-α co-treated C2C12 myotubes led to the upregulation of the VDR, SIRT1, and SIRT3 expression. Additionally, 1,25VD3 treatment restored the reduced levels of myogenic markers MyoD and MyoG induced by IFN-γ/TNF-α. Furthermore, the interactive roles of VDR, SIRT1, and SIRT3 in the regulation of MyoD and MyoG activities, which are essential for muscle growth and regeneration, have been demonstrated. Garcia et al. showed that upregulation of VDR by 1,25VD3 in C2C12 skeletal muscle cells promotes muscle cell differentiation by increasing MyoD activity [47], and Braga et al. showed that incubation of skeletal muscle-derived stem cells with 1,25VD3 induces increased the expression of MyoD and MyoG [48]. Another study found that SIRT3 depletion in C2C12 myoblasts impaired terminal differentiation and reduced the expression of MyoG, MyoD, and SIRT1, indicating SIRT3’s role in myoblast differentiation regulation and suggesting that crosstalk between SIRT3 and SIRT1 collaborates in fine-tuning the differentiation process [49]. These findings suggested a collaborative function of VDR, SIRT1, and SIRT3 in regulating muscle growth and myogenesis by modulating the expression of MyoD and MyoG.

Additionally, 1,25VD3 + IFN-γ/TNF-α treatment restored AMPK and AKT phosphorylation levels, which were lowered by IFN-γ/TNF-α treatment (Figure 5). Moreover, 1,25VD3 activation of VDR, SIRT1, and AMPK protects against fat accumulation, oxidative stress, and mitochondrial dysfunction in skeletal muscle cells and in vivo [24,30]. SIRT1 and AMPK form a reciprocal positive regulatory loop, with AMPK activating SIRT1 through increased NAD+ levels and SIRT1 activating AMPK by deacetylating LKB1 [38,50]. Similarly, SIRT3, a mitochondrial sirtuin, directly and indirectly regulates AMPK by deacetylating LKB1 and other AMPK-regulating proteins, leading to AMPK phosphorylation and activation. The decrease in AMPK phosphorylation observed in the muscle tissue of SIRT3 knockout mice indicates that SIRT3 plays a positive role in AMPK activation [28,51,52]. Upon 1,25VD3 binding to VDR, coactivators are recruited, leading to increased AKT phosphorylation. Furthermore, SIRT1 deacetylates AKT, enhancing its binding to PIP3 and its subsequent activation, whereas SIRT3 regulates ROS-mediated AKT activation [53]. These findings highlight the intricate interplay between vitamin D/VDR signaling and SIRT1 and SIRT3 in the modulation of AMPK, AKT, and related cellular processes.

FoxO3a transcriptional activity is finely regulated by post-translational modifications such as phosphorylation and deacetylation, which affect its activation and deactivation [54]. Our study revealed that 1,25VD3 treatment on IFN-γ/TNF-α co-treated C2C12 myotubes restored the VDR/SIRT1/SIRT3 axis, leading to increased FoxO3 phosphorylation, inhibiting its transcriptional activity, and preventing muscle protein degradation through the FoxO3a/MAFbx/MuRF1 pathway (Figure 6). VDR promotes FoxO3a deacetylation through SIRT1 and SIRT3 expression, whereas AMPK activation induces FoxO3a phosphorylation, leading to reduced transcriptional activity [55,56,57]. PI3K stimulation enhances AKT activity, causing the phosphorylation of FoxO3 proteins. This leads to their retention in the cytoplasm, preventing nuclear translocation and the subsequent inhibition of MuRF1 and MAFbx transcription [58,59]. Vitamin D deficiency in rats results in elevated protein breakdown and MAFbx/MuRF1 expression, which can be reversed by restoring vitamin D levels [60]. Motta et al. highlighted the significant role of SIRT1 in regulating FOXO3a activity through deacetylation upon binding to FOXO3a [61]. We showed that 1,25VD3 administration upregulated VDR, SIRT1, and SIRT3 and restored AMPK and AKT activation, leading to improved muscle development, myotube diameter, and MyHC expression by reducing FoxO3a transcriptional activity. This underscores the importance of 1,25VD3 in maintaining skeletal muscle health by regulating the activity of FoxO3a and its downstream targets.

1,25VD3 treatment upregulates the VDR/SIRT1/SIRT3 axis, protecting against IFN-γ/TNF-α-induced apoptosis and promoting cell survival by downregulating cleaved-caspase 3, cleaved PARP, and Bax activity, inhibiting both mitochondrial-dependent and nuclear-dependent apoptosis pathways [62,63]. PARP, an enzyme involved in DNA repair in muscle cells, is cleaved and inactivated by active caspases during apoptosis or DNA damage, potentially contributing to cell death and muscle degeneration. Elevated VDR levels inhibit PARP-induced cell apoptosis through the VDR/PARP1 pathway, whereas SIRT1/SIRT3 deacetylate PARP, obstruct ADP-ribose transfer, and prevent DNA damage-induced cell death [56,57,62,64,65]. Treatment with SIRT1 overexpression or resveratrol (a SIRT1 activator) leads to PARP-1 deacetylation [65]. Furthermore, resveratrol activates AMPK in SIRT1-deficient cells and reduced AMPK activity suppresses resveratrol-induced SIRT1 activation, suggesting that AMPK is the initial trigger in the SIRT1-PARP loop [57]. Increased apoptosis-related myocyte loss involves Bax and caspases activating caspase-dependent or caspase-independent pathways, culminating in muscle fiber degradation [66,67]. Bax initiates cell death by releasing cytochrome c and activating caspase 3. SIRT3 attenuates Bax levels and activates SOD2 to counteract oxidative stress [68]. Upon activation, Bax relocates to the outer mitochondrial membrane, assembles into oligomers, permeabilizes the membrane, and releases death-promoting factors, such as cytochrome c [63]. SIRT1 prevents apoptosis through Ku70 interactions, deacetylating it during caloric restriction, and reducing stress-induced apoptosis by sequestering Bax from the mitochondria [18,69]. The osteoblast anti-apoptotic effects of 1,25VD3 stem from non-genomic VDR/PI3K/AKT pathway activation, culminating in reduced caspase activities [70]. Therefore, modulation of the VDR/SIRT1/SIRT3 axis through 1,25VD3 treatment is a multilayered defensive strategy against apoptosis induction.

Activation of the VDR/SIRT1/SIRT3 axis through 1,25VD3 leads to increased expression of OXPHOS-related mRNA and preservation of mtDNA copy number (Figure 7). Prolonged vitamin D deficiency reduces muscle health by decreasing mtDNA content and the expression of critical biogenesis-related factors (for example, PGC-1α, NRF1, NRF2, PPARα, and COXIV). Restoring optimal vitamin D levels not only alleviates myopathy symptoms but also enhances muscle mitochondrial oxidative capacity [71,72]. Sirtuin deacetylases control protein acetylation/deacetylation within the mitochondria, influencing mitochondrial function [73]. Decreased nuclear NAD+ levels decrease SIRT1 activity, causing a specific decline in mitochondria-encoded components during oxidative phosphorylation [71]. Decreased SIRT1 activity during aging is associated with decreased oxidative capacity and ATP synthesis. Inhibition of AMPK disrupts SIRT1-mediated PGC-1α deacetylation, affecting both respiratory activity and the shift to fatty acid oxidation metabolism [74,75]. SIRT3 activation, facilitated by PGC-1α under energy stress, enhances mitochondrial function and OXPHOS [18]. Recent research has emphasized that SIRT3 deficiency causes extensive acetylation at over 400 mitochondrial sites that are involved in all aspects of mitochondrial biological functions, including OXPHOS [75]. This leads to mitochondrial hyperacetylation and reduced PGC-1α targets (NRF-1 and mtTFA), whereas SIRT3 overexpression increases mitochondrial DNA content [75]. SIRT3 deacetylates and activates subunits of the OXPHOS complex (complexes I, II, III, IV, and V) in the electron transport chain and significantly influences mitochondrial biogenesis, gene expression, and activation of oxidative phosphorylation components [75]. Together these data imply that activating VDR, SIRT1, and SIRT3 through 1,25VD3 can not only prevent IFN-γ/TNF-α-induced muscle atrophy and apoptosis but also improve OXPHOS and mitochondrial biogenesis in C2C12 myotubes.

## 5. Conclusions

**Figure 8:**
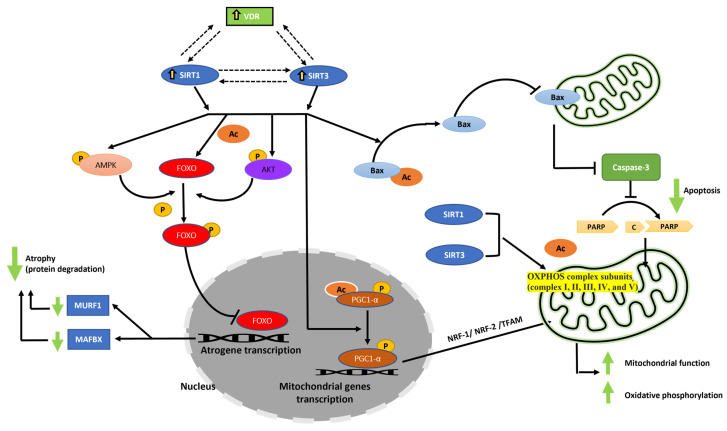
Schematic diagram by which Vitamin D and the VDR/SIRT1/SIRT3 axis might promote hypertrophy and inhibit apoptosis in skeletal muscle cells. Ac, Acetyl group; P, Phosphate group, C, Cleaved-PARP.

As shown in Figure 8 as a schematic diagram, this study highlights the essential role of 1,25VD3 in modulating VDR, SIRT1, and SIRT3 expression, where VDR fosters a synergistic VDR/SIRT1/SIRT3 axis through interdependent interactions. These upregulated proteins activate AMPK and AKT, enhancing FoxO3a phosphorylation and inhibiting protein degradation and muscle atrophy. Additionally, elevated VDR, SIRT1, and SIRT3 suppress PARP, Caspase 3, and Bax, inhibiting mitochondrial and nuclear apoptosis. Moreover, compelling evidence highlights the capability of VDR, SIRT1, and SIRT3 to enhance PGC-1α expression as a pivotal catalyst for optimal mitochondrial function [24,74,75,76]. This elevation yields increased levels of vital proteins crucial for mitochondrial health and facilitates OXPHOS [18,75]. SIRT1 and SIRT3 play pivotal roles in nuclear-mitochondrial interactions. SIRT1 orchestrates the regulation of PGC-1α and FoxO3 activities, impacting PGC-1α gene expression within the nucleus. Beyond substrate deacetylation, SIRT3’s influence extends to nuclear-mitochondrial crosstalk [75]. This interplay involves key factors like FoxO3, PGC-1α, AMPK, SIRT1, and SIRT3, collectively driving mitochondrial biogenesis through transcriptional initiation and activation via phosphorylation and deacetylation processes.

1,25VD3, VDR, SIRT1, and SIRT3 form an interconnected relationship, with VDR influencing the expression of SIRT1 and SIRT3 and vice versa. Activation of the VDR/SIRT1/SIRT3 axis by 1,25VD3 leads to modifications in downstream proteins, including FoxO3a, AMPK, AKT, Bax, Caspase 3, and PARP. These modifications inhibit muscle protein breakdown and prevent cell death through the mitochondrial and nuclear pathways. Increased levels of VDR, SIRT1, and SIRT3 enhance the expression of PGC1α, a regulator of mitochondrial function, promoting oxidative phosphorylation and improving mitochondrial function.

Finally, our study successfully validated VDR expression in C2C12 cells, providing insights into the impact of 1,25VD3 on C2C12 myotubes through VDR/SIRT1/3 axis activation. This study shows that Vitamin D and the VDR/SIRT1/SIRT3 axis may promote hypertrophy and inhibit apoptosis in skeletal muscle cells. In addition, it provides experimental evidence that vitamin D has the potential to be developed as a therapeutic or preventive agent against sarcopenia through the stimulation of the VDR/SIRT1/SIRT3 axis. However, uncertainties remain about how these findings from a muscle cell line translate to in vivo muscle. Previous studies have discussed the significant contrast in VDR expression levels among mature muscle, satellite cells, and myoblasts, indicating higher detectability of VDR in cell models compared to whole muscle tissue [77,78]. In our study, we primarily investigated C2C12 myotubes. However, we recognize the need for further research in mature muscle tissue and alternative cell models to substantiate and refine our findings, ultimately enhancing the reliability and applicability of our conclusions. While some reviews on human studies lean towards a positive impact of vitamin D in treating or preventing sarcopenia in older adults, it is essential to carefully assess both positive [79] and negative [80] evidence when tailoring supplementation approaches for individuals with sarcopenia.

## Data Availability

Data are contained within the article and Supplementary Materials.

## Supplementary Materials

The following supporting information can be downloaded at: https://www.mdpi.com/article/10.3390/nu15224714/s1, Table S1: Primer sequences used in Real-time qPCR; Table S2: Antibody list used in Western blot; Figure S1: The Effects of 1,25VD3 on myotube diameters, VDR, SIRT1, SIRT3, MyoD, MyoG protein expression, and AMPK/AKT Activation; Figure S2: Effects of IFN-γ/TNF-α treatment on protein expression of VDR, SIRT1, SIRT3, MyHC I, and MyHC II.; Figure S3: Effects of 1,25VD3 on preventing the downregulation of VDR, SIRT1, SIRT3, MyHC I, MyHC II, MyoD, and MyoG protein expression induced by IFN-γ/TNF-α treatment.
