# Supplementary material for: Vitamin D3 Exerts Beneficial Effects on C2C12 Myotubes through Activation of the Vitamin D Receptor (VDR)/Sirtuins (SIRT)1/3 Axis
Source: Nutrients. 2023 Nov 7;15(22):4714. doi: 10.3390/nu15224714 (PMC10674540; doi:10.3390/nu15224714)
Supplement: Supplementary file 1 [file nutrients-15-04714-s001.zip › nutrients-2647955-supplementary.pptx]

## Slide 1
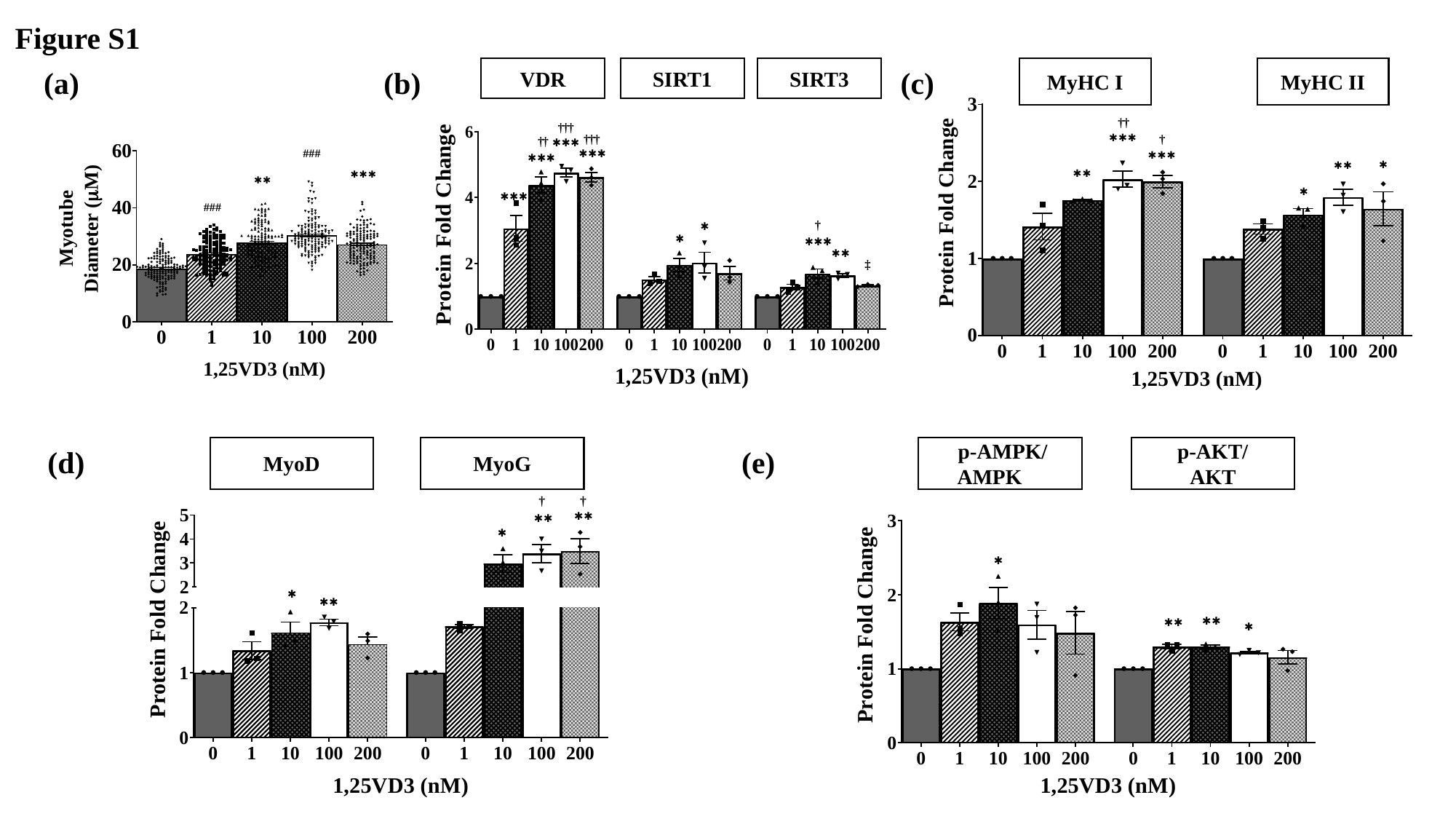

Figure S1
(a)
(b)
VDR
SIRT1
SIRT3
(c)
MyHC I
MyHC II
(d)
MyoD
MyoG
(e)
 p-AMPK/
AMPK
p-AKT/
AKT

## Slide 2
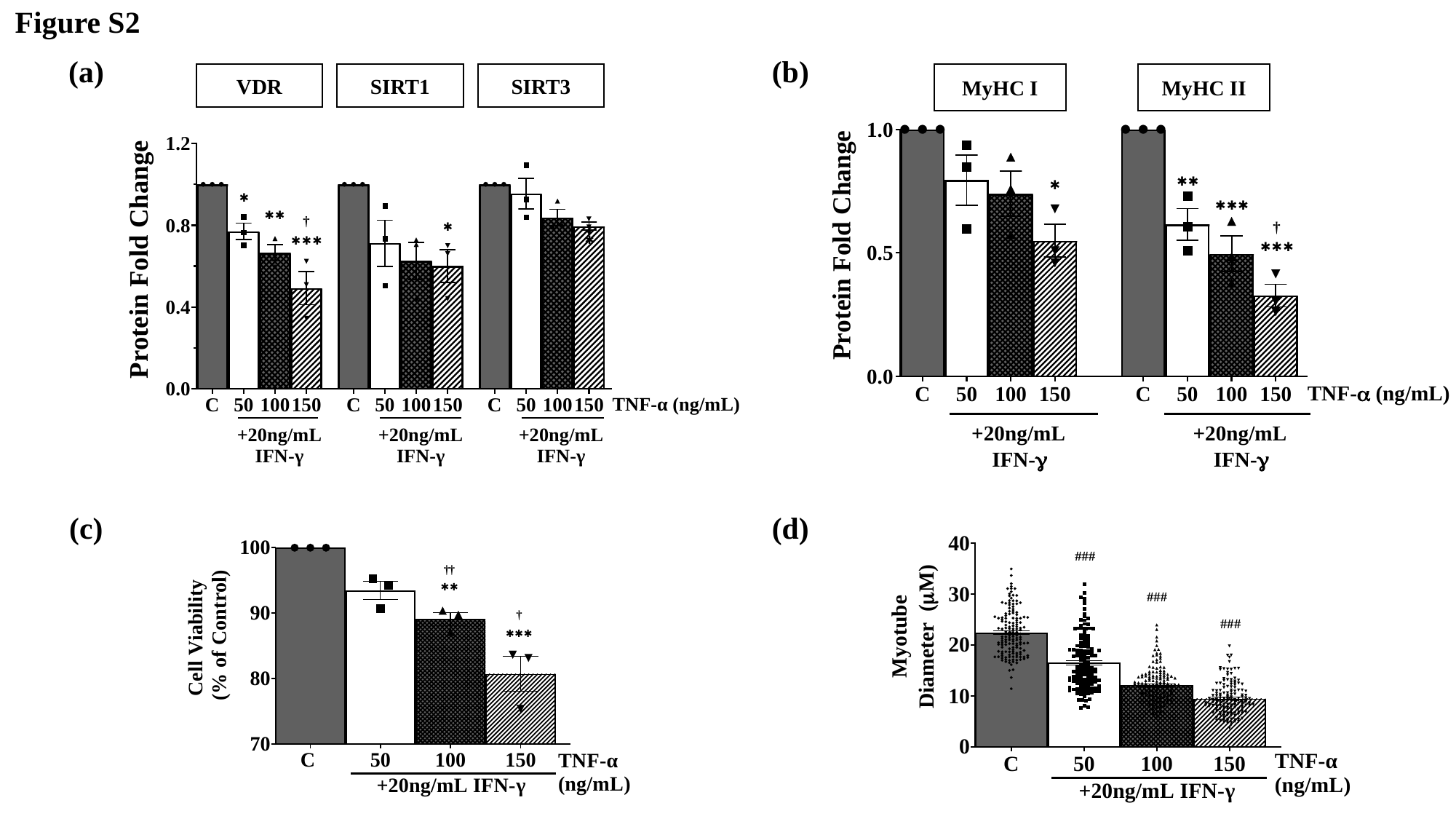

Figure S2
(a)
(b)
VDR
SIRT1
SIRT3
MyHC I
MyHC II
(c)
(d)

## Slide 3
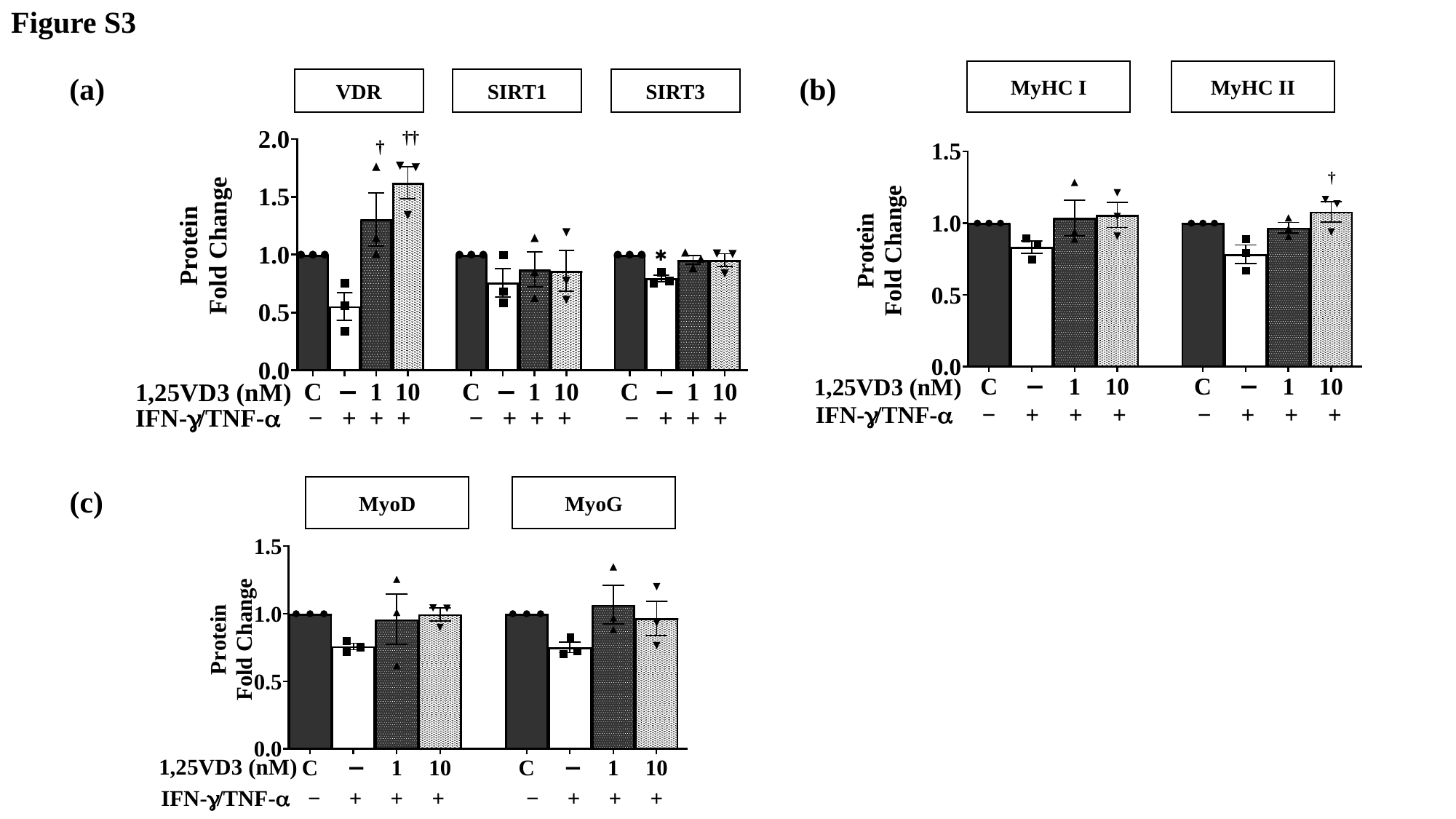

Figure S3
MyHC I
MyHC II
(a)
(b)
VDR
SIRT1
SIRT3
(c)
MyoD
MyoG
