# Supplementary material for: Vitamin D3 Exerts Beneficial Effects on C2C12 Myotubes through Activation of the Vitamin D Receptor (VDR)/Sirtuins (SIRT)1/3 Axis
Source: Nutrients. 2023 Nov 7;15(22):4714. doi: 10.3390/nu15224714 (PMC10674540; doi:10.3390/nu15224714)
Supplement: Supplementary file 1 [file nutrients-15-04714-s001.zip › nutrients-2647955_Supplementary Table S1 and S2.pdf]

Table S1: Primer sequences used in Real-time qPCR

No.	Primer Name	Sequence (5' -> 3')	*(°C)*
**1**	β-ACTIN-Forward	GGCTCTTTTCCAGCCTTCCT	*60.0*
**2**	β-ACTIN-Reverse	AATGCCTGGGTACATGGTGG	*60.0*
**3**	VDR-Forward	CACCTGGCTGATCTTGTCAGT	*60.0*
**4**	VDR-Reverse	CTGGTCATCAGAGGTGAGGTC	*60.0*
**5**	SIRT1-Forward	AGTTCCAGCCGTCTCTGTGT	*60.0*
**6**	SIRT1-Reverse	CTCCACGAACAGCTTCACAA	*60.0*
**7**	SIRT3-Forward	TGCCTGCAAGGTTCCTACTC	*60.0*
**8**	SIRT3-Reverse	CGAGGACTCAGAACGAACGG	*60.0*
**9**	FoxO3a-Forward	TCACACTACGGCAACCAGAC	*61.0*
**10**	FoxO3A-Reverse	TGGGCAGCAAAGGACATCAT	*61.0*
**11**	Atrogin1 (MAFbx)-Forward	TCACACTACGGCAACCAGAC	*61.0*
**12**	Atrogin1 (MAFbx)-Reverse	TGGGCAGCAAAGGACATCAT	*61.0*
**13**	MuRF1 -Forward	GTTTGACACCCTCTACGCCA	*61.0*
**14**	MuRF1 -Reverse	TTGAGAGGAAGGTAGCCCCT	*61.0*
**15**	MyHC I (MYH7)-Forward	AGATCCGAAAGCAACTGGAG	*60.0*
**16**	MyHC I (MYH7)-Reverse	CTGCCTTGATCTGGTTGAAC	*60.0*
**17**	MyHC IIa (MYH2)-Forward	GCAGAGACCGAGAAGGAG	*60.0*
**18**	MyHC IIa (MYH2)-Reverse	CTTTCAAGAGGGACACCATC	*60.0*
**19**	MyHC IIX (MYH1)-Forward	GCGACAGACACCTCCTTCAAG	*61.0*
**20**	MyHC IIX (MYH1)-Reverse	TCCAGCCAGCCAGCGATG	*61.0*
**21**	MyHC IIb (MYH4)-Forward	CAACTGAGTGAAGTGAAGACC	*60.0*
**22**	MyHC IIb (MYH4)-Reverse	AGCTGAGAAACCATAGCGTC	*60.0*
**23**	m-NDUFB8-Forward	CGCCAAGAAGTATAACATGCGAG	*60.0*
**24**	m-NDUFB8-Reverse	CCTCTCATGCTGTGATCGGTTG	*60.0*
**25**	m-ATP5A1-Forward	TCGGCCATTTTGTGCCAGTC	*60.0*
**26**	m-ATP5A1-Reverse	AGTCGAGTGTTAGAGGCATGG	*60.0*

**27**	m-COX1-Forward	AACCATAGGGCACCAATGATAC	*60.0*
**28**	m-COX1-Reverse	GGATGGCATCAGTTTTAAGTCC	*60.0*
**29**	m-SDHB-Forward	ACCCCTTCTCTGTCTACCG	*60.0*
**30**	m-SDHB-Reverse	AATGCTCGCTTCTCCTTGTAG	*60.0*
**31**	m-UQCR2-Forward	ATGCCACCTTCTACCGTCCTTC	*60.0*
**32**	m-UQCR2-Reverse	GTTTCCACTCGCTGCCATTGAC	*60.0*

Table S2: Antibody list

No.	Antibodies	Type	Size	Dilution	Catalogue Number
**1**	Goat anti-Rabbit IgG Polyclonal	HRP conjugate	-	1:10000	ADI-SAB-300
**2**	Goat anti-Mouse IgG Heavy and Light Chain	HRP conjugate	-	1:10000	A90-116P
**3**	Vitamin D Receptor/VDR (D-6)	Mouse Monoclonal Antibody	48kDa	1:500	sc-13133
**4**	Skeletal Muscle Myosin (F59)	Mouse Monoclonal antibody	200kDa	1:1000	sc-32732
**5**	Anti-Myosin (Skeletal, Slow)	Mouse Monoclonal antibody	200kDa	1:5000	M8421
**6**	Anti-SIRT1 [19A7AB4]	Mouse Monoclonal antibody	110kDa	1:10000	ab110304
**7**	SIRT3 (D22A3)	Rabbit Monoclonal antibody	28kDa	1:1000	5490S
**8**	Anti-MyoD (5.8A)	Mouse Monoclonal antibody	45kDa	1:1000	sc-32758
**9**	Myogenin (F5D)	Mouse Monoclonal antibody	35kDa	1:1000	sc-12732
**10**	Phospho-AMPKα (Thr172)	Mouse Monoclonal antibody	60kDa	1:1000	2531S
**11**	AMPKα	Mouse Monoclonal antibody	60kDa	1:1000	2532S
**12**	Akt1/2/3 (5C10)	Mouse Monoclonal antibody	62kDa	1:1000	sc-81434
**13**	p-Akt1/2/3 (B-5)	Mouse Monoclonal antibody	62/56/60k Da	1:1000	sc-271966
**14**	FoxO3a (75D8)	Rabbit Monoclonal antibody	82-97kDa	1:1000	2497S
**15**	Phospho-FoxO3a (Ser253)	Rabbit Monoclonal antibody	97kDa	1:1000	9466S
**16**	MAFbx (F-9)	Mouse Monoclonal antibody	45kDa	1:500	sc-166806
**17**	MuRF1 (C-11)	Mouse Monoclonal antibody	40kDa	1:500	sc-398608
**18**	Caspase-3	Rabbit Monoclonal antibody	17, 19, 35kDa	1:1000	9662S
**19**	PARP	Rabbit Monoclonal antibody	89, 116kDa	1:1000	9532S
**20**	Bax (B-9)	Mouse Monoclonal antibody	23kDa	1:1000	sc-7480
